# Transjugular intrahepatic portosystemic shunt for hepatic sinusoidal obstruction syndrome associated with consumption of Gynura segetum

**DOI:** 10.1186/s12876-021-01599-7

**Published:** 2021-01-10

**Authors:** Lijie Zhang, Qing Li, Joyman Makamure, Dan Zhao, Ziyi Liu, Chuansheng Zheng, Bin Liang

**Affiliations:** Department of Radiology, Hubei Key Laboratory of Molecular Imaging, Union Hospital, Tongji Medical College, Huazhong University of Science and Technology, 1277 Jiefang Road, Wuhan, 430022 China

**Keywords:** Hepatic sinusoidal obstruction syndrome, Transjugular intrahepatic portosystemic shunt, Gynura segetum

## Abstract

**Background:**

To evaluate the efficacy and safety of transjugular intrahepatic portosystemic shunt (TIPS) on hepatic sinusoidal obstruction syndrome (HSOS) associated with consumption of Gynura segetum (GS).

**Methods:**

We retrospectively reviewed 9 consecutive patients with GS-related HSOS who were refractory to supportive treatment and underwent TIPS at our institution between January 2014 and September 2019. The patients were evaluated for safety and efficacy, including TIPS complications and changes in portosystemic pressure gradient (PPG), ascites, total bilirubin, liver size and portal vein diameter.

**Results:**

TIPS procedures were performed successfully in the 9 patients, and no technically-related complications due to the TIPS procedure were recorded. The PPG was improved by TIPS in all patients (mean PPG before TIPS, 30.4 ± 5.2 vs. 13.0 ± 4.1 mm Hg post-TIPS, *P* = 0.008). One patient who was lost to follow-up, whereas the remaining 8 patients survived with a median follow-up period of 12 months (range 5–39 months). Although the total bilirubin was significantly increased 5–7 days after TIPS compared with that before the procedure (3.57 ± 1.58 vs. 4.82 ± 2.06 mg/dl, *P* = 0.017), it returned to baseline levels at 1-month follow-up (3.53 ± 2.72 vs. 4.82 ± 2.06 mg/dl, *P* = 0.401). The patients experienced complete resolution or noticeable reduction of ascites (*P* < 0.001), significant reduction of liver size (16.7 ± 2.2 vs. 13.7 ± 1.7 cm, *P* = 0.018), and significant enlargement of the portal trunk (10.7 ± 2.5 vs. 13.4 ± 2.4 mm, *P* = 0.017) after TIPS compared to the pre-TIPS state.

**Conclusion:**

TIPS may offer a potentially useful treatment for the GS-related HSOS.

## Background

Hepatic sinusoidal obstruction syndrome (HSOS), previously called hepatic veno-occlusive disease, refers to obstruction of hepatic venous outflow at the level of the central or sub-lobular hepatic veins, or both [1, 2]. The syndrome is clinically characterized by hepatomegaly, ascites, weight gain and jaundice [3]. Multiple risk factors have been observed to be related to HSOS. One major etiology of HSOS in China is the consumption of pyrrolizidine alkaloid-containing Gynura segetum (GS) [4], Tusanqi [5]. Tusanqi is occasionally erroneously ingested because it is mistaken for a Chinese traditional herb with a similarly sounding name, Sanqi [6]. GS-induced liver injury involves fibrous obliteration and destruction of the central veins and sub-lobular venules, leading to cirrhosis [7].

Current therapies for the GS-related HSOS focus on supportive care and anticoagulation. Unfortunately, the prognosis of patients with severe HSOS remains poor [8, 9]. Transjugular intrahepatic portosystemic shunt (TIPS) has been used to treat HSOS which is refractory to supportive therapies and anticoagulation [10]. The rationale for TIPS in the treatment of HSOS is similar to that of Budd-Chiari syndrome, in which the portosystemic shunt relieves hepatic congestion and thus increases hepatic artery perfusion and improves hepatic function [11]. However, the efficacy of TIPS for GS-related HSOS remains controversial [8, 9].

We report the preliminary efficacy and safety of TIPS for HSOS associated with consumption of GS.

## Methods

### Patients

A retrospective analysis of 9 consecutive patients (6 males and 3 females was conducted; age range 49–78 years; median age, 59 years) who were diagnosed with GS-related HSOS and received TIPS at Union Hospital, Tongji Medical College, Huazhong University of Science and Technology from January 2014 to July 2019. Based on the diagnostic criteria for GS-related HSOS in China [7], the patients were diagnosed as GS-related HSOS when they had (1) a history of recent GS intake, (2) clinical manifestation of hepatomegaly, jaundice and ascites, (3) laboratory values showing liver function abnormalities, especially an increase in total bilirubin, and (4) characteristic findings of HSOS on CT images, including hepatomegaly, hepatic parenchyma with heterogeneous hypoattenuation, patchy heterogeneous enhancement with or without thin or non-visualized hepatic veins. Patients were excluded from this study if they had any other potential risk factors associated with the development of HSOS, such as pre-existing liver disease and bone marrow transplantation [3]. They were also unsuitable for or unwilling to liver transplantation.

This study was approved by the Ethics Committee of Tongji Medical College, Huazhong University of Science and Technology (IORG No: IORG0003571) and performed in accordance with local and national laws and the principles of the declaration of Helsinki. Written informed consent was obtained for all patients.

### Pre-TIPS workup and medication

Prior to TIPS, all patients underwent complete history and physical examination with particular attention to liver disease and patients’ performance status. A contrast-enhanced CT scan was performed on all patients to assess the hepatic and vascular anatomy and to select the most proper portosystemic shunting tract. Baseline laboratory values, including liver function tests, complete blood count, creatinine, and coagulation profile were also obtained. Hepatic encephalopathy (HE) was graded based on the West Haven Grading System [12].

Of the 9 patients, 7 received at least 2 weeks of supportive measures including fluid and sodium restriction, diuretics and therapeutic paracenteses, combined with anticoagulation with low molecular weight heparin. The remaining two subjects received supportive measures alone as obvious gastroesophageal varices were noted on pre-procedure CT scan, but the symptoms did not improve or became worse. After TIPS, all patients received extended anticoagulation with oral warfarin until complete remission.

### TIPS procedure

**Fig. 1:**
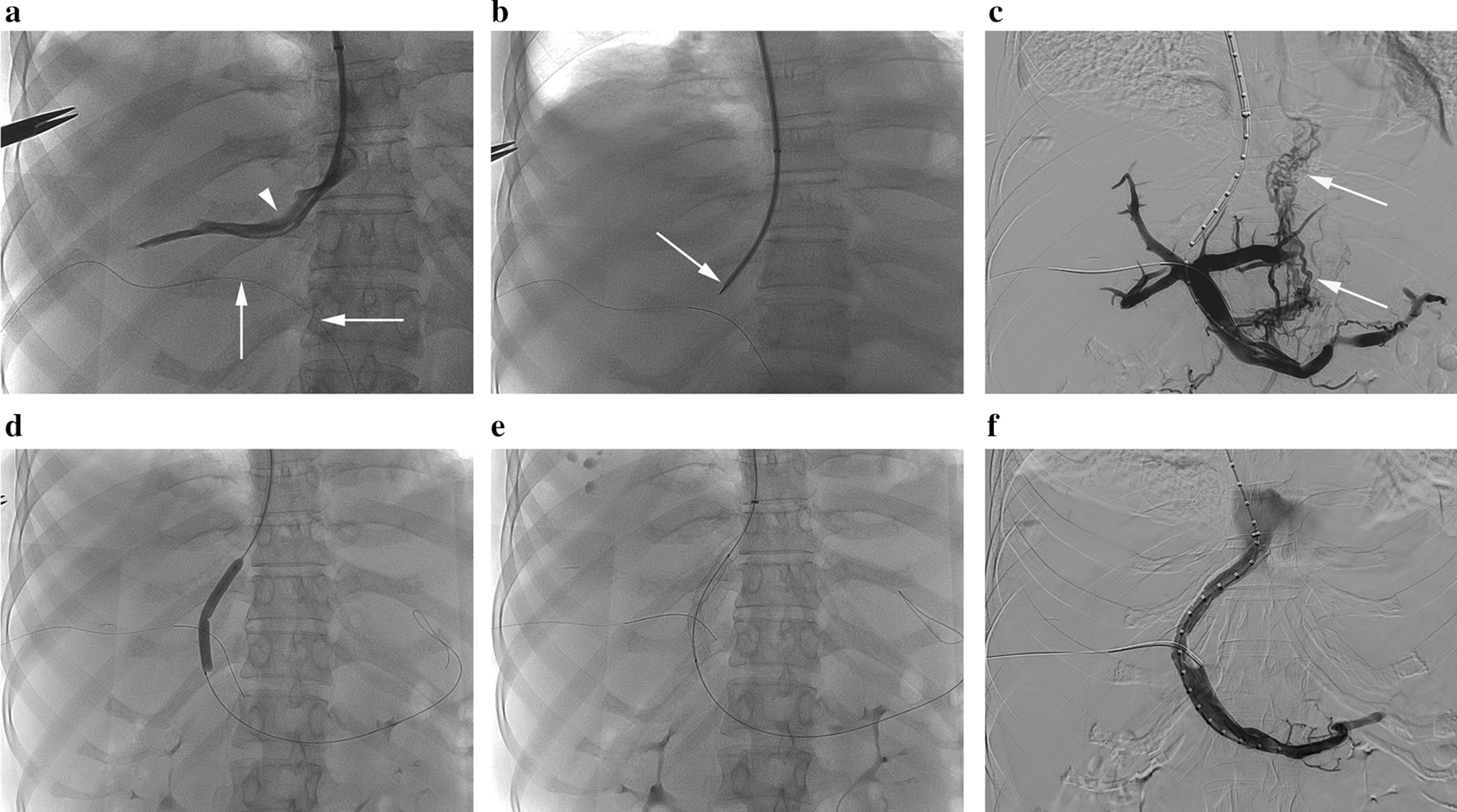
TIPS procedures for GS-related HSOS. **a** Right hepatic venogram through a catheter positioned in the right hepatic vein showed the relationship of the right hepatic vein (arrowhead) with the right portal vein (vertical arrow) and portal vein trunk (horizontal arrow) into which a 0.014-in guide wire was percutaneously inserted. **b** Puncture of the right portal vein with a Rösch-Uchida TIPS needle (arrow). **c** Portogram through a calibrated pigtail catheter positioned in the splenic vein confirmed the successful access to the portal vein. Note the persistent opacification of the gastroesophageal varices (arrow) due to the portal hypertension. **d** Dilatation of the parenchymal tract with a 6-mm-diameter balloon. **e** Deploying a bare stent and subsequently a covered stent inside the bare stent across the portosystemic tract. Distending the stents to an 8 mm diameter if necessary. **f** Post-TIPS portogram demonstrated flow through the TIPS shunt, with no opacification of any gastroesophageal varices

The TIPS procedure was performed under the guidance of ultrasound and digital subtraction angiography (Artis Zee Celling, Siemens Medical Solutions, Muenchen, Germany). In summary, venous access was attained through the right internal jugular vein, then afterwards the right or middle hepatic vein was catheterized. A standard Rösch-Uchida TIPS set (Cook Medical, Bloomington, IN, US) was used to create a parenchymal tract between the hepatic vein and the intrahepatic portion of the portal vein. In some patients in whom access to the portal vein by transhepatic puncture proved difficult, percutaneous insertion of a 0.014-in wire into the portal system would be used to provide access. After measurement of pressures in the portal vein and the right atrium, the tract was dilated with balloon catheters, and then a bare stent (Bard E-Luminexx® Vascular Stent, C. R. Bard, Inc, Karlsruhe, Germany) followed by a stent-graft (Viabahn, W. L. Gore & Associates, Inc, Flagstaff, Arizona, US) were deployed in series to line the tract and dilated to achieve an internal diameter. All stents were 8 mm in size. The length of bare stent was selected according to the general rule of thumb, measuring the length from the entry site in the portal vein to the inferior vena cava and then adding 1–2 cm to the length. The added length of bare metal was meant to be in the portal vein. The covered stents were 5 or 10 cm in length, with its distal portion extending slightly into the portal vein. No further embolization of gastroesophageal varices was required in all patients. The portosystemic pressure gradient (PPG) was measured after the creation of portosystemic shunts (Fig. 1a–f). Technical success of the TIPS was defined as the successful creation of a shunt between the hepatic vein and intrahepatic branch of the portal vein.

### Complications and efficacy evaluation

The complications related to the TIPS procedure, including hepatic venography, nontarget needle puncture and stent malposition, were assessed during periprocedural period. The incidence of post-TIPS complications, such as shunt failure, development of HE, liver failure and other severe adverse events, were also assessed during the periprocedural and follow-up periods.

All patients underwent repeat clinical and laboratory assessment 5–7 days after TIPS, and then were followed up at 1 month and every 3–6 months thereafter. The follow-up CT scan was performed for assessment of stent patency as well as hepatic and vascular changes. The maximal liver height was measured on coronal CT image and the diameter of the portal vein trunk was measured on axial CT images by two experienced radiologists in consensus. Patients were followed up until death, loss to follow-up or the end of the study (October, 2019).

### Statistical analysis

Statistical analysis was performed by using SPSS 17.0 software (SPSS, Chicago, Ill). Data was presented as mean ± standard deviation and as range and median unless otherwise stated. Difference in PPG, bilirubin, ascites and CT findings before and after TIPS were compared with Mann–Whitney U-test. A *P* value of less than 0.05 was considered statistically significant.

## Results

### Patients’ characteristics

**Fig. 2:**
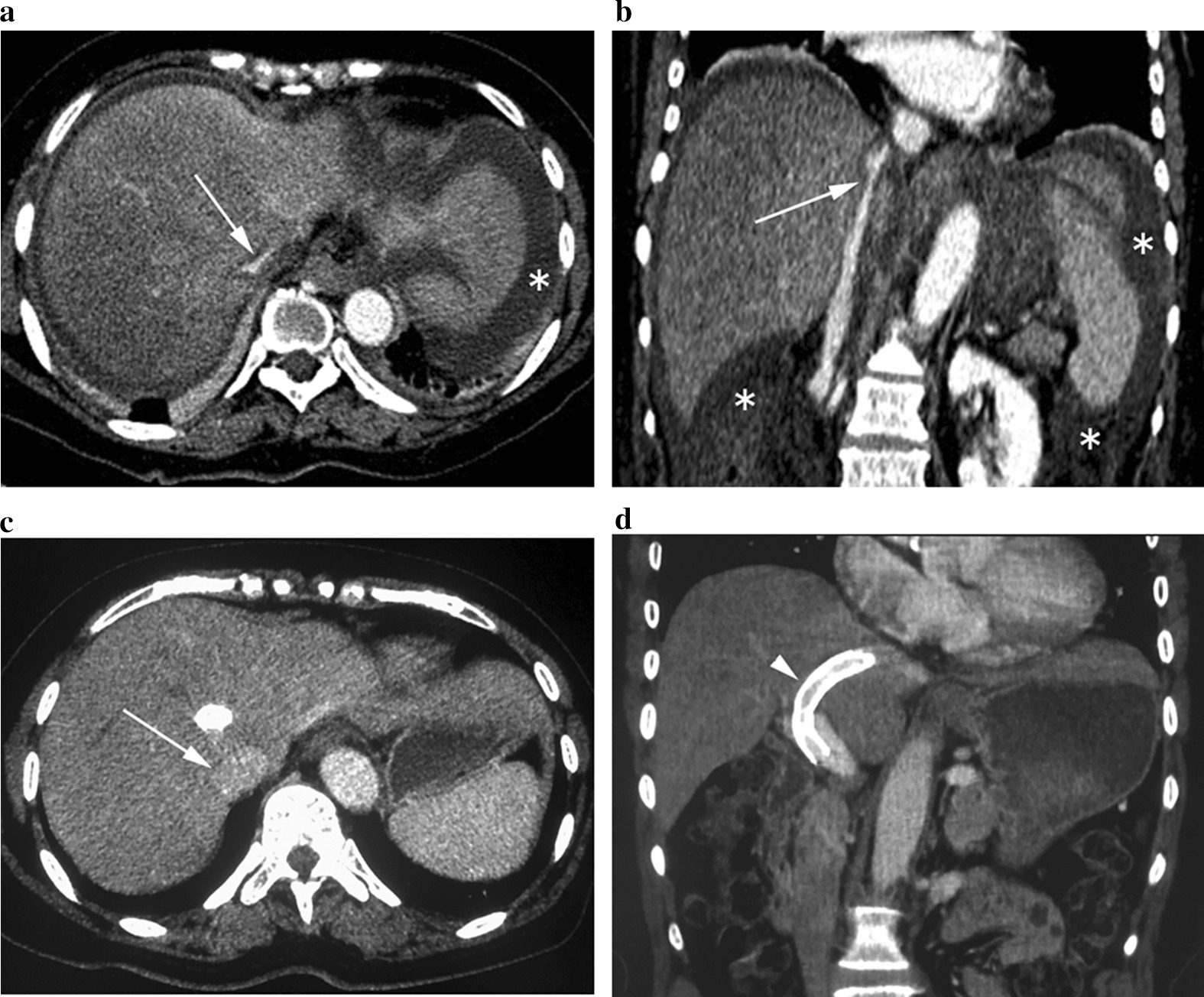
CT findings of GS-related HSOS before and after TIPS. Contrast-enhanced axial (**a**) and coronal (**b**) CT images showed patchy heterogeneous enhancement of the liver, with increased size of the liver, decreased size of the hepatic veins and hepatic segment of the inferior vena cava (arrow), and massive ascites (asterisk). Follow-up axial (**c**) and coronal (**d**) CT images demonstrated that the liver enhancement returns to homogeneous, with normal size of the liver, hepatic veins and inferior vena cava (arrow), and complete resorption of ascites. Note the patent TIPS stents (arrowhead)

Table 1 summarizes the patients’ characteristics before the TIPS procedure. The included patients consisted of 3 women and 6 men. The median age was 59 years (range 49–78 years). All patients had the history of GS intake and presented with hepatomegaly, jaundice and ascites, with characteristic findings of HSOS on CT images (Fig. 2a, b). Notably, all the patients developed gastroesophageal varices on DSA images, but none had variceal bleeding. One patient developed symptoms 2 years after intermittent intake of GS, whereas the other 8 patients had a median time frame from GS consumption to symptom onset of 5 months (range 1–10 months). Only 1 patient presented with grade I HE.

**Table 1:** Demographic and clinical characteristics of the 9 patients

Case	Age (years)	Time to onset after Tusanqi intake (m)	Bilirubin (μmol/L)	ALT (U/L)	PT seconds over control	Albumin (g/L)	CR (μmol/L)	Ascites (grade)	HE	Child Pugh score	MELD score
1	50	5	37.1	149	6.2	29.6	61.7	+++	0	11	17
2	66	24	28.7	43	0	29.0	116.0	+++	0	8	13
3	78	3	75.8	41	11.3	29.9	127.0	+++	I	13	26
4	55	1	92.4	64	5.9	25.1	130.0	+++	0	12	24
5	58	1.5	73.6	98	1.6	32.6	85.2	+++	0	10	16
6	54	6	87.7	615	7.3	32.8	103.0	+++	0	12	23
7	49	6	38.0	27	3.8	30.2	70.2	+++	0	9	17
8	66	5	66.3	27	0	26.2	107.0	+++	0	11	16
9	62	10	24.5	133	0	26.0	120.0	+++	0	9	17

*ALT* alanine transaminase, *PT* prothrombin time, *HE* hepatic encephalopathy, *CR* creatinine

+, mild; ++, moderate; +++, severe

### TIPS technique and complications

TIPS procedures were performed as planned in all patients. The 8-mm stents were deployed in 8 patients, and 10-mm stents were used in 1 patient. After TIPS, the mean PPG was significantly reduced from 30.4 ± 5.2 mmHg (range 24–40 mmHg, median, 30 mmHg,) to 13.0 ± 4.1 mmHg (range 6–19 mm Hg, median, 13 mmHg); the difference was significant (*P* = 0.008).

The technical success rate of TIPS was 100%, without any TIPS procedure-related complications. With regard to the post-TIPS complications, more severe HE (grade III) was observed in the patient who had grade I HE before TIPS. The patient responded to treatment with lactulose and aspartate-ornithine, and his condition improved after the initiation of treatment. Thrombocytopenia was detected in another patient during the periprocedural period, but he improved with platelet transfusion. No shunt failure or liver failure was noted in all patients during the periprocedural and follow-up periods.

### Efficacy of TIPS for HSOS

Only 1 patient was lost to follow-up, and the remaining 8 patients were followed up for a median of 12 months (range 5–39 months). All the follow-up patients survived at the end of the follow-up period without additional liver transplantation.

Table 2 shows the efficacy of TIPS in the HSOS patients. Ascites was reduced in 5 patients and disappeared in 4 patients at 5–7 days after TIPS, and at 1-month follow-up only 1 patient had mild ascites. Although the level of total bilirubin demonstrated a significant increase at 5–7 days after TIPS as compared to the pre-TIPS state in all patients (82.8 ± 35.0 μmol/L vs. 60.76 ± 26.9 μmol/L, *P* = 0.017), it returned to near baseline level 1 month later (60.0 ± 46.2 μmol/L vs. 60.76 ± 26.9 μmol/L, *P* < 0.001). The maximal liver height after TIPS was significantly decreased compared to that before TIPS (13.7 ± 1.7 cm vs 16.7 ± 2.2 cm, *P* = 0.018), whereas the diameter of the portal vein trunk was significantly increased after TIPS compared to that before TIPS (13.4 ± 2.4 mm vs 10.7 ± 2.5, *P* = 0.017) (Fig. 2a–d). All the implanted stents remained patent on the follow-up CT scans.

**Table 2:** Changes in PPG, CR, BUN, bilirubin, ascites, and CT findings after TIPS

Case	PPG (mmHg)		CR(μmol/L)		BUN(mmol/L)		Bilirubin (μmol/L)			Ascites (grade)			Liver height (cm)		Portal vein diameter (mm)	
	Before	After	Before	Day 5–7	Before	Day 5–7	Before	Day 5–7	Follow-up	BEFORE	Day 5–7	Follow-up	Before	Follow-up	Before	Follow-up
1	26	6	61.7	49.8	4.0	1.5	37.1	58.3	52.4	+++	−	−	18.6	13.1	12.1	16.7
2	28	8	116.0	128.5	9.3	5.5	28.7	41.8	23.1	+++	+	−	17.2	17.2	14.2	14.1
3	24	13	127.0	124.2	8.1	6.3	75.8	112.9	168.5	+++	++	+	12.3	12	10.6	12.4
4	35	14	130.0	86.4	10.6	5.4	92.4	108.9	39.6	+++	−	−	18.2	13.7	9.8	13.9
5	34	19	85.2	72.3	3.8	2.0	73.6	107.9	57.8	+++	+	−	17.0	13.5	7.0	10.2
6	40	16	103.0	84.2	7.8	3.3	87.7	125.1	64.8	+++	++	−	19.2	14.8	13.0	16.4
7	30	10	70.2	63.8	5.3	2.7	38	23.9	NA	+++	+	NA	18.9	NA	13.6	NA
8	31	15	107.0	87.4	11.7	6.1	66.3	62.1	47.6	+++	−	−	15.5	12.2	7.6	10.8
9	25	12	120.0	62.9	5.4	4.8	24.5	39.7	26.4	+++	−	−	15.8	13.2	10.9	12.3

*PPG* portosystemic pressure gradient, *CR* creatinine, *BUN* blood urea nitrogen, *NA* not available

+, mild; ++, moderate; +++, severe; −, absent

## Discussion

TIPS has not been recommended for HSOS by the most recent clinical practice guidelines [13, 14] because the procedure did not show survival benefit in patients with HSOS after bone marrow transplantation [15–18]. However, it is still unclear whether TIPS can be used to treat HSOS associated with GS. In this study, we demonstrated that TIPS may offer a potentially useful treatment in GS-related HSOS.

Previous studies evaluating the efficacy of TIPS in GS-related HSOS have yielded conflicting results. Wang et al. [8] investigated the effect of anticoagulation and TIPS on survival of patients with GS-related HSOS and found that TIPS failed to improve the overall survival rate. This outcome could possibly have been due to the flaws in the study design. In this study [8], TIPS was performed in patients whose symptoms did not improve or worsened after 2 weeks of anticoagulation therapy. In addition, the patients’ characteristics were not compared between different treatment groups. It is likely that the HSOS patients in the TIPS group had more severe illness than those of the anticoagulation group, which led to the aforementioned results. In contrast, another retrospective study by Zhuge et al. [9] compared the efficacy of anticoagulation with or without TIPS and supportive treatment for GS-related HSOS and found that anticoagulation noticeably improved the survival rate and TIPS further improved the prognosis of patients who did not respond to anticoagulation therapy. Given the fact that HSOS patients presented with gastroesophageal varices and had the risk of variceal bleeding, anticoagulants before TIPS should be administered with caution. In our study, two patients did not receive pre-TIPS anticoagulation therapy due to the presence of obvious gastroesophageal varices. All patients were refractory to medications and then received TIPS followed by anticoagulation therapy. These patients responded positively to the treatment and achieved a favorable prognosis. The ascites quickly resolved within several days after TIPS procedure. The size of the liver and portal vein returned to normal on follow up. Despite a transient impairment of the liver function after TIPS, the patients recovered with the relevant supportive treatment. All patients survived at the end of the follow-up period. These data suggest that TIPS may represent an effective therapy for GS-related HSOS, with satisfactory patient survival as well as clinical and laboratory parameters. This is different from the high mortality rate after TIPS for HSOS following bone marrow transplantation [15–18].

Although a large number of procedure-related and post-procedural complications of TIPS have been identified, it can be safely performed in patients with GS-related HSOS [13, 19, 20]. The procedure-related complications include issues related to venous access, wedged hepatic venography, transhepatic needle puncture, stent placement, etc. In the prior study by Zhuge et al. [9], one of the 27 GS-related HSOS patients who received TIPS treatment died from intraperitoneal hemorrhage during the TIPS procedure. In this study, no procedure-related complications were noted, which may be attributed to the careful preprocedural evaluation and procedure technique. The most challenging part of TIPS creation is arguably the transvenous access from the hepatic vein to the portal vein. For patients in whom the puncture of portal vein was difficult, technique for portal vein targeting such as percutaneous insertion of a microwire into the portal system was employed to improve the success rate of the shunt tract puncture. On the other hand, the postprocedural complications of TIPS include recurrence of ascites, variceal bleeding, HE, liver failure, death, etc. The first two complications are considered to be related to insufficient portosystemic shunting, whereas the latter two are related to excessive shunting. In the previous study by Wang et al. [8], five of the 14 patients who were refractory to anticoagulation and received TIPS treatment died from complications or multiple organ failure during follow-up. Unfortunately, further details about the complications were not provided. In this study, one of the 9 patients developed severe HE one month after TIPS. We believe that the exacerbation of HE may be related to severe liver dysfunction before TIPS and excessive portosystemic shunting.

Two advices can be drawn from our study for the TIPS procedure of GS-related HSOS. One is related to the gastroesophageal varices. Although gastroesophageal varices were noted on DSA images in all patients, no further embolization of the gastroesophageal varices was needed after the TIPS creation. No patients experienced variceal bleeding before and after the TIPS. The other is the change in PPG. The post-procedural PPG decreased from 30.4 ± 5.2 to 13.0 ± 4.1 mmHg with implantation of the 8-mm stents and the patients achieved satisfactory clinical outcomes, which suggest that a target PPG of 50% of the initial gradient seems to be sufficient for the HSOS-induced ascites. Excessive shunting may increase the risk of HE.

There are several limitations in this study. Firstly, we did not perform liver biopsy on these patients; therefore no information regarding the pre- and post-procedural histopathology was obtained. Additional studies based on the biopsy evidence are needed to understand the mechanism of action of TIPS for the GS-related HSOS. Secondly, because the VIATORR® stent-graft (Gore® Medical, Arizona USA) was not commercially available in our institution, we used the combination of a bare stent and a covered stent for the TIPS creation. Our results demonstrated that all the stents remained patent during the follow-up period, which suggests that the combined stents may offer an alternative to VIATORR® stent-graft for TIPS application. Finally, the sample size was relatively small due to the low incidence rate of GS-related HSOS, which may result in an inevitable bias.

## Conclusions

In conclusion, our study demonstrated that TIPS may present a safe and effective treatment for GS-related HSOS refractory to supportive therapies. Future prospective clinical trials with larger sample sizes and longer follow-up periods are required to determine the precise efficacy of TIPS in this setting.

## Data Availability

All data and materials are not available in this study, and are available from the corresponding author on reasonable requests.

## Abbreviations

TIPS: Transjugular intrahepatic portosystemic shunt
HSOS: Hepatic sinusoidal obstruction syndrome
GS: Gynura segetum
